# Dibromido(di-2-pyridylamine-κ^2^ *N*,*N*′)mercury(II)

**DOI:** 10.1107/S1600536808038129

**Published:** 2008-11-20

**Authors:** Khadijeh Kalateh, Ali Norouzi, Amin Ebadi, Roya Ahmadi, Vahid Amani

**Affiliations:** a Islamic Azad University, Shahr-e-Rey Branch, Tehran, Iran; b Islamic Azad University, Izeh Branch, Khozestan, Iran; c Department of Chemistry, Islamic Azad University, Kazeroon Branch, Fars, Iran

## Abstract

In the mol­ecule of the title compound, [HgBr_2_(C_10_H_9_N_3_)], the Hg^II^ atom is four-coordinated in a distorted tetra­hedral configuration by two N atoms from the chelating di-2-pyridylamine ligand and by two Br atoms. In the crystal structure, inter­molecular N—H⋯Br hydrogen bonds link the mol­ecules into centrosymmetric dimers. There are π–π contacts between the pyridine rings [centroid–centroid distances = 3.9662 (5) and 3.9321 (4) Å]. There also exists a C—H⋯π contact between the pyridine CH group and a pyridine ring.

## Related literature

For related literature, see: Ahmadi *et al.* (2008 ▶); Kalateh *et al.* (2008 ▶); Khavasi *et al.* (2008 ▶); Tadayon Pour *et al.* (2008 ▶); Yousefi, Rashidi Vahid *et al.* (2008 ▶); Yousefi, Tadayon Pour *et al.* (2008 ▶). For related structures, see: Xie *et al.* (2004 ▶); Hughes *et al.* (1985 ▶).
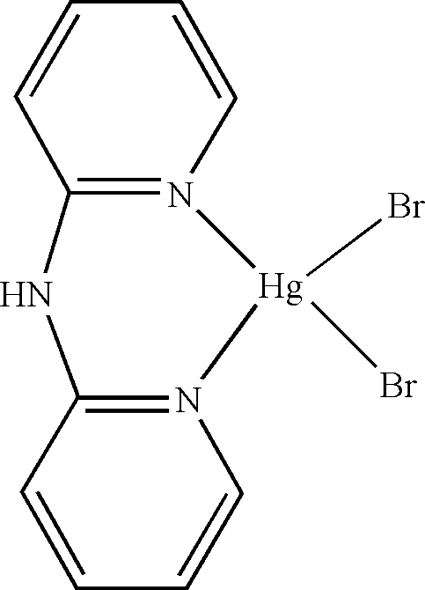


## Experimental

### 

#### Crystal data

[HgBr_2_(C_10_H_9_N_3_)]
*M* *_r_* = 531.59
Triclinic, 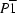

*a* = 8.1284 (16) Å
*b* = 8.7645 (18) Å
*c* = 9.912 (2) Å
α = 113.45 (3)°
β = 98.41 (3)°
γ = 97.79 (3)°
*V* = 626.1 (3) Å^3^
*Z* = 2
Mo *K*α radiation
μ = 18.65 mm^−1^
*T* = 120 (2) K
0.40 × 0.35 × 0.25 mm

#### Data collection

Bruker SMART CCD area-detector diffractometer
Absorption correction: numerical (shape of crystal determined optically) (*X-SHAPE* and *X-RED*; Stoe & Cie, 2005 ▶)*T*_min_ = 0.016, *T*_max_ = 0.080
7839 measured reflections
3350 independent reflections
3234 reflections with *I* > 2σ(*I*)
*R*_int_ = 0.087

#### Refinement

*R*[*F* ^2^ > 2σ(*F* ^2^)] = 0.053
*wR*(*F* ^2^) = 0.140
*S* = 1.15
3350 reflections
145 parameters
H-atom parameters constrained
Δρ_max_ = 4.33 e Å^−3^
Δρ_min_ = −6.54 e Å^−3^

### 

Data collection: *SMART* (Bruker, 1998 ▶); cell refinement: *SAINT* (Bruker, 1998 ▶); data reduction: *SAINT*; program(s) used to solve structure: *SHELXTL* (Sheldrick, 2008 ▶); program(s) used to refine structure: *SHELXTL*; molecular graphics: *ORTEP-3 for Windows* (Farrugia, 1997 ▶); software used to prepare material for publication: *WinGX* (Farrugia, 1999 ▶).

## Supplementary Material

Crystal structure: contains datablocks I, global. DOI: 10.1107/S1600536808038129/hk2575sup1.cif

Structure factors: contains datablocks I. DOI: 10.1107/S1600536808038129/hk2575Isup2.hkl

Additional supplementary materials:  crystallographic information; 3D view; checkCIF report

## Figures and Tables

**Table:** 

Br1—Hg1	2.5106 (11)
Br2—Hg1	2.5549 (11)
N1—Hg1	2.301 (7)
N3—Hg1	2.350 (7)

**Table:** 

N1—Hg1—N3	81.1 (2)
N1—Hg1—Br1	109.13 (17)
N3—Hg1—Br1	117.16 (17)
N1—Hg1—Br2	125.41 (17)
N3—Hg1—Br2	96.23 (18)
Br1—Hg1—Br2	119.68 (3)

**Table 2:** Hydrogen-bond geometry (Å, °)

*D*—H⋯*A*	*D*—H	H⋯*A*	*D*⋯*A*	*D*—H⋯*A*
N2—H2*A*⋯Br2^i^	0.86	2.62	3.472 (3)	170
C2—H2⋯*Cg*3^ii^	0.93	3.20	3.587 (3)	107

Symmetry codes: (i) 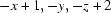; (ii) 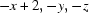. *Cg*3 is the  centroid of the N3/C6–C10 ring.

**Fig. 1.**
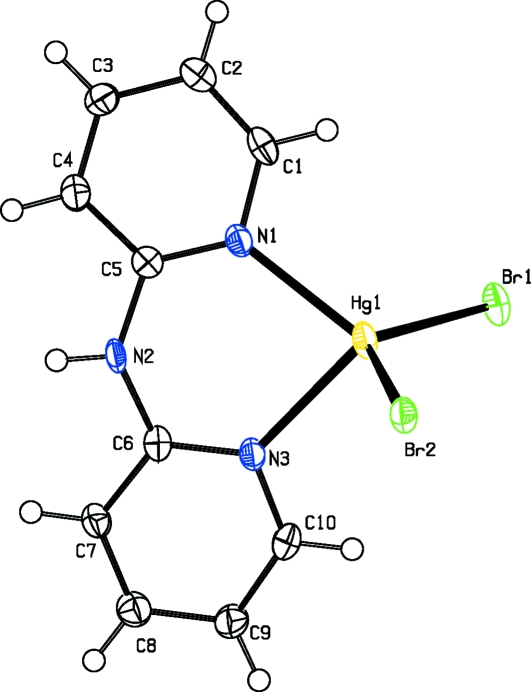
The molecular structure of the title molecule, with the atom-numbering scheme. Displacement ellipsoids are drawn at the 50% probability level.

**Fig. 2.**
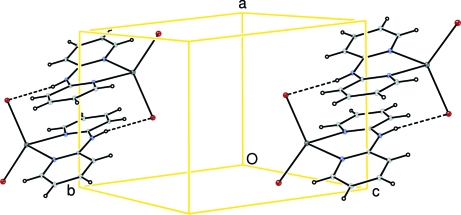
A partial packing diagram of the title compound.

## supplementary crystallographic information

### Comment

Recently, we reported the syntheses and crystal structures of [Hg(4,4'-dmbpy) I_2_], (II), (Yousefi, Tadayon Pour *et al.*, 2008), [Hg(5,5'-dmbpy)I_2_], (III), (Tadayon Pour, *et al.*, 2008), [Hg(dmphen)I_2_], (IV), (Yousefi, Rashidi Vahid *et al.*, 2008), {[HgCl(dm4bt)]_2_(µ-Cl)_2_}, (V), (Khavasi *et al.*, 2008), [Hg(6-mbpy)Cl_2_], (VI), (Ahmadi *et al.*, 2008) and [{HgBr(4,4'-dmbpy)}_2_(µ-Br)_2_], (VII), (Kalateh *et al.*, 2008) [where 4,4'-dmbpy is 4,4'-dimethyl-2,2'-bipyridine, 5,5'-dmbpy is 5,5'-dimethyl-2,2'- bipyridine, 6-mbpy is 6-methyl-2,2'-bipyridine, dmphen is 4,7-diphenyl-1,10- phenanthroline and dm4bt is 2,2'-dimethyl-4,4'-bithiazole]. There are two Hg^II^ complexes, with formula, [Hg(N—N)Br_2_], such as [Hg(TPA)Br_2_], (VIII), (Xie *et al.*, 2004) and [Hg(TPD)Br_2_], (IX), (Hughes *et al.*, 1985) [where TPA is tris(2-pyridyl)amine and TPD is *N*,*N*,*N*',*N*'-Tetramethyl-*o*-phenylenediamine] have been synthesized and characterized by single-crystal X-ray diffraction methods. We report herein the synthesis and crystal structure of the title compound, (I).

In the title compound, (Fig. 1), the Hg^II^ atom is four-coordinated in a distorted tetrahedral configuration by two N atoms from di-2-pyridylamine and two Br atoms. The Hg-Br and Hg-N bond lengths and angles (Table 1) are within normal ranges, as in (VIII).

In the crystal structure, intermolecular N-H···Br hydrogen bonds link the molecules into centrosymmetric dimers  (Fig. 2), in which they may be effective in the stabilization of the structure. The π-π contacts between the pyridine rings Cg2···Cg2^i^ and Cg2···Cg3^ii^ [symmetry codes: (i) x, -y, 2 - z, (ii) 1 - x,- y, 2 - z, where Cg2 and Cg3 are centroids of the rings A (N1/C1-C5) and B (N3/C6-C10), respectively] may further stabilize the structure, with centroid-centroid distances of 3.9662 (5) %A and 3.9321 (4) %A, respectively. There also exists a  C—H···π contact (Table 1) between the pyridine CH group and pyridine ring.

### Experimental

For the preparation of the title compound, (I), a solution of di-2-pyridylamine (0.25 g, 1.43 mmol) in methanol (20 ml) was added to a solution of HgBr_2_ (0.51 g, 1.43 mmol) in acetonitrile (20 ml) and the resulting colorless solution was stirred for 20 min at 313 K. This solution was left to evaporate slowly at room temperature. After one week, colorless prismatic crystals of the title compound were isolated (yield; 0.55 g, 72.3%).

### Refinement

H atoms were positioned geometrically, with N-H = 0.86 Å (for NH) and C-H = 0.93 Å for aromatic H and constrained to ride on their parent atoms, with U_iso_(H) = 1.2U_eq_(C,N). The highest and lowest peaks are located 0.69 Å and 0.78 Å from Hg1 atom, respectively.

### Crystal data

**Table:**

[HgBr_2_(C_10_H_9_N_3_)]	*Z* = 2
*M**_r_* = 531.59	*F*_000_ = 480
Triclinic, *P*1	*D*_x_ = 2.820 Mg m^−^^3^
Hall symbol: -P 1	Mo *K*α radiation λ = 0.71073 Å
*a* = 8.1284 (16) Å	Cell parameters from 1657 reflections
*b* = 8.7645 (18) Å	θ = 2.3–29.2º
*c* = 9.912 (2) Å	µ = 18.65 mm^−^^1^
α = 113.45 (3)º	*T* = 120 (2) K
β = 98.41 (3)º	Prism, colorless
γ = 97.79 (3)º	0.40 × 0.35 × 0.25 mm
*V* = 626.1 (3) Å^3^	

### Data collection

**Table:**

Bruker SMART CCD area-detector diffractometer	3350 independent reflections
Radiation source: fine-focus sealed tube	3234 reflections with *I* > 2σ(*I*)
Monochromator: graphite	*R*_int_ = 0.087
*T* = 120(2) K	θ_max_ = 29.2º
φ and ω scans	θ_min_ = 2.3º
Absorption correction: numerical(shape of crystal determined optically)	*h* = −11→11
*T*_min_ = 0.016, *T*_max_ = 0.080	*k* = −11→10
7839 measured reflections	*l* = −12→13

### Refinement

**Table:**

Refinement on *F*^2^	Secondary atom site location: difference Fourier map
Least-squares matrix: full	Hydrogen site location: inferred from neighbouring sites
*R*[*F*^2^ > 2σ(*F*^2^)] = 0.053	H-atom parameters constrained
*wR*(*F*^2^) = 0.140	*w* = 1/[σ^2^(*F*_o_^2^) + (0.0882*P*)^2^ + 2.3955*P*] where *P* = (*F*_o_^2^ + 2*F*_c_^2^)/3
*S* = 1.15	(Δ/σ)_max_ = 0.059
3350 reflections	Δρ_max_ = 4.33 e Å^−^^3^
145 parameters	Δρ_min_ = −6.54 e Å^−^^3^
Primary atom site location: structure-invariant direct methods	Extinction correction: none

### Special details

**Table:**

Geometry. All e.s.d.'s (except the e.s.d. in the dihedral angle between two l.s. planes) are estimated using the full covariance matrix. The cell e.s.d.'s are taken into account individually in the estimation of e.s.d.'s in distances, angles and torsion angles; correlations between e.s.d.'s in cell parameters are only used when they are defined by crystal symmetry. An approximate (isotropic) treatment of cell e.s.d.'s is used for estimating e.s.d.'s involving l.s. planes.
Refinement. Refinement of *F*^2^ against ALL reflections. The weighted *R*-factor *wR* and goodness of fit *S* are based on *F*^2^, conventional *R*-factors *R* are based on *F*, with *F* set to zero for negative *F*^2^. The threshold expression of *F*^2^ > σ(*F*^2^) is used only for calculating *R*-factors(gt) *etc*. and is not relevant to the choice of reflections for refinement. *R*-factors based on *F*^2^ are statistically about twice as large as those based on *F*, and *R*- factors based on ALL data will be even larger.

### Fractional atomic coordinates and isotropic or equivalent isotropic displacement parameters (Å^2^)

**Table:**

	*x*	*y*	*z*	*U*_iso_*/*U*_eq_	
Hg1	0.28140 (3)	0.22806 (4)	0.84793 (3)	0.02226 (15)	
Br1	0.03376 (10)	0.29535 (11)	0.71493 (10)	0.0259 (2)	
Br2	0.58403 (10)	0.34148 (10)	0.83842 (9)	0.0219 (2)	
N1	0.2053 (9)	0.1755 (9)	1.0425 (8)	0.0195 (12)	
N2	0.3018 (9)	−0.0818 (8)	0.9915 (8)	0.0190 (12)	
H2A	0.3426	−0.1362	1.0395	0.023*	
N3	0.3006 (9)	−0.0591 (8)	0.7601 (8)	0.0192 (12)	
C1	0.1347 (11)	0.2988 (10)	1.1369 (11)	0.0239 (15)	
H1	0.1098	0.3845	1.1096	0.029*	
C2	0.0991 (11)	0.3023 (11)	1.2678 (11)	0.0248 (16)	
H2	0.0516	0.3881	1.3288	0.030*	
C3	0.1363 (11)	0.1728 (11)	1.3080 (10)	0.0247 (15)	
H3	0.1151	0.1727	1.3977	0.030*	
C4	0.2037 (11)	0.0462 (11)	1.2157 (10)	0.0225 (14)	
H4	0.2279	−0.0412	1.2407	0.027*	
C5	0.2355 (9)	0.0519 (10)	1.0815 (9)	0.0178 (13)	
C6	0.3175 (9)	−0.1477 (10)	0.8430 (9)	0.0172 (13)	
C7	0.3535 (10)	−0.3113 (10)	0.7834 (10)	0.0215 (14)	
H7	0.3679	−0.3693	0.8437	0.026*	
C8	0.3671 (11)	−0.3850 (11)	0.6355 (10)	0.0249 (15)	
H8	0.3886	−0.4940	0.5944	0.030*	
C9	0.3485 (12)	−0.2952 (11)	0.5481 (10)	0.0262 (16)	
H9	0.3575	−0.3414	0.4479	0.031*	
C10	0.3158 (11)	−0.1340 (12)	0.6165 (10)	0.0248 (16)	
H10	0.3034	−0.0731	0.5587	0.030*	

### Atomic displacement parameters (Å^2^)

**Table:**

	*U*^11^	*U*^22^	*U*^33^	*U*^12^	*U*^13^	*U*^23^
Hg1	0.0242 (2)	0.0212 (2)	0.0290 (2)	0.00845 (13)	0.00573 (13)	0.01720 (14)
Br1	0.0239 (4)	0.0253 (4)	0.0341 (4)	0.0070 (3)	0.0012 (3)	0.0194 (3)
Br2	0.0237 (4)	0.0208 (4)	0.0267 (4)	0.0057 (3)	0.0061 (3)	0.0151 (3)
N1	0.023 (3)	0.016 (3)	0.019 (3)	0.006 (2)	0.001 (2)	0.008 (2)
N2	0.026 (3)	0.015 (3)	0.022 (3)	0.007 (2)	0.004 (2)	0.013 (2)
N3	0.024 (3)	0.016 (3)	0.018 (3)	0.005 (2)	0.002 (2)	0.009 (2)
C1	0.025 (4)	0.015 (3)	0.032 (4)	0.005 (3)	0.003 (3)	0.011 (3)
C2	0.024 (3)	0.020 (3)	0.030 (4)	0.007 (3)	0.007 (3)	0.009 (3)
C3	0.029 (4)	0.024 (4)	0.022 (3)	0.006 (3)	0.009 (3)	0.010 (3)
C4	0.026 (3)	0.021 (3)	0.024 (4)	0.005 (3)	0.003 (3)	0.012 (3)
C5	0.014 (3)	0.017 (3)	0.020 (3)	0.001 (2)	0.002 (2)	0.007 (3)
C6	0.013 (3)	0.016 (3)	0.020 (3)	−0.002 (2)	−0.002 (2)	0.009 (3)
C7	0.024 (3)	0.017 (3)	0.024 (4)	0.004 (3)	0.004 (3)	0.010 (3)
C8	0.027 (4)	0.022 (4)	0.023 (4)	0.005 (3)	0.002 (3)	0.008 (3)
C9	0.037 (4)	0.021 (4)	0.017 (3)	0.005 (3)	0.003 (3)	0.006 (3)
C10	0.030 (4)	0.029 (4)	0.020 (3)	0.008 (3)	0.004 (3)	0.015 (3)

### Geometric parameters (Å, °)

**Table:**

Br1—Hg1	2.5106 (11)	C5—N1	1.327 (10)
Br2—Hg1	2.5549 (11)	C5—N2	1.393 (10)
N1—Hg1	2.301 (7)	C6—N3	1.342 (10)
N2—H2A	0.8600	C6—N2	1.384 (10)
N3—Hg1	2.350 (7)	C6—C7	1.408 (11)
C1—C2	1.360 (13)	C7—C8	1.376 (12)
C1—N1	1.376 (11)	C7—H7	0.9300
C1—H1	0.9300	C8—C9	1.389 (13)
C2—C3	1.398 (13)	C8—H8	0.9300
C2—H2	0.9300	C9—C10	1.385 (12)
C3—C4	1.368 (12)	C9—H9	0.9300
C3—H3	0.9300	C10—N3	1.342 (11)
C4—C5	1.410 (11)	C10—H10	0.9300
C4—H4	0.9300		
			
N1—Hg1—N3	81.1 (2)	C2—C3—H3	119.9
N1—Hg1—Br1	109.13 (17)	C3—C4—C5	118.2 (8)
N3—Hg1—Br1	117.16 (17)	C3—C4—H4	120.9
N1—Hg1—Br2	125.41 (17)	C5—C4—H4	120.9
N3—Hg1—Br2	96.23 (18)	N1—C5—N2	121.7 (7)
Br1—Hg1—Br2	119.68 (3)	N1—C5—C4	122.9 (7)
C1—N1—Hg1	114.3 (5)	N2—C5—C4	115.4 (7)
C5—N1—Hg1	128.2 (6)	N3—C6—N2	121.6 (7)
C5—N1—C1	117.2 (7)	N3—C6—C7	121.6 (7)
C6—N2—C5	135.0 (7)	N2—C6—C7	116.9 (7)
C6—N2—H2A	112.5	C8—C7—C6	119.6 (8)
C5—N2—H2A	112.5	C8—C7—H7	120.2
C6—N3—Hg1	126.5 (5)	C6—C7—H7	120.2
C10—N3—Hg1	115.6 (6)	C7—C8—C9	119.4 (8)
C10—N3—C6	117.4 (7)	C7—C8—H8	120.3
C2—C1—N1	123.6 (8)	C9—C8—H8	120.3
C2—C1—H1	118.1	C8—C9—C10	117.2 (8)
N1—C1—H1	118.3	C8—C9—H9	121.4
C1—C2—C3	117.9 (8)	C10—C9—H9	121.4
C1—C2—H2	121.0	N3—C10—C9	124.8 (8)
C3—C2—H2	121.1	N3—C10—H10	117.5
C4—C3—C2	120.2 (8)	C9—C10—H10	117.7
C4—C3—H3	120.0		
			
C1—N1—Hg1—N3	−166.6 (6)	C6—C7—C8—C9	1.3 (12)
C5—N1—Hg1—N3	20.2 (6)	C7—C8—C9—C10	−0.3 (13)
C1—N1—Hg1—Br1	−50.8 (6)	C8—C9—C10—N3	−0.1 (14)
C5—N1—Hg1—Br1	136.0 (6)	N2—C5—N1—C1	178.0 (7)
C1—N1—Hg1—Br2	102.0 (5)	C4—C5—N1—C1	−2.2 (11)
C5—N1—Hg1—Br2	−71.1 (7)	N2—C5—N1—Hg1	−9.0 (10)
C10—N3—Hg1—N1	168.0 (6)	C4—C5—N1—Hg1	170.8 (6)
C6—N3—Hg1—N1	−20.2 (6)	C2—C1—N1—C5	1.8 (12)
C6—N3—Hg1—Br1	−127.2 (6)	C2—C1—N1—Hg1	−172.2 (7)
C10—N3—Hg1—Br1	61.0 (6)	N3—C6—N2—C5	16.3 (13)
C6—N3—Hg1—Br2	104.8 (6)	C7—C6—N2—C5	−164.2 (8)
C10—N3—Hg1—Br2	−67.0 (6)	N1—C5—N2—C6	−16.8 (13)
N1—C1—C2—C3	−0.2 (13)	C4—C5—N2—C6	163.3 (8)
C1—C2—C3—C4	−1.0 (13)	C9—C10—N3—C6	−0.4 (13)
C2—C3—C4—C5	0.6 (12)	C9—C10—N3—Hg1	172.2 (7)
C3—C4—C5—N1	1.0 (12)	N2—C6—N3—C10	−179.2 (7)
C3—C4—C5—N2	−179.1 (7)	C7—C6—N3—C10	1.4 (11)
N3—C6—C7—C8	−1.8 (11)	N2—C6—N3—Hg1	9.2 (10)
N2—C6—C7—C8	178.7 (7)	C7—C6—N3—Hg1	−170.3 (5)

### Hydrogen-bond geometry (Å, °)

**Table:**

*D*—H···*A*	*D*—H	H···*A*	*D*···*A*	*D*—H···*A*
N2—H2A···Br2^i^	0.86	2.62	3.472 (3)	170
C2—H2···Cg3^ii^	0.93	3.20	3.587 (3)	107

Symmetry codes: (i) −*x*+1, −*y*, −*z*+2; (ii) −*x*+2, −*y*, −*z*.
